# Spectroscopic and Imaging Technologies Combined with Machine Learning for Intelligent Perception of Pesticide Residues in Fruits and Vegetables

**DOI:** 10.3390/foods14152679

**Published:** 2025-07-30

**Authors:** Haiyan He, Zhoutao Li, Qian Qin, Yue Yu, Yuanxin Guo, Sheng Cai, Zhanming Li

**Affiliations:** 1 School of Grain Science and Technology, Jiangsu University of Science and Technology, Zhenjiang 212100, Chinaguoyuanxin@just.edu.cn (Y.G.); 2 Fujian Putian Sea 100 Food Co., Ltd., Putian 351111, China

**Keywords:** pesticide residue, spectral technology, imaging, machine learning, deep learning

## Abstract

Pesticide residues in fruits and vegetables pose a serious threat to food safety. Traditional detection methods have defects such as complex operation, high cost, and long detection time. Therefore, it is of great significance to develop rapid, non-destructive, and efficient detection technologies and equipment. In recent years, the combination of spectroscopic techniques and imaging technologies with machine learning algorithms has developed rapidly, providing a new attempt to solve this problem. This review focuses on the research progress of the combination of spectroscopic techniques (near-infrared spectroscopy (NIRS), hyperspectral imaging technology (HSI), surface-enhanced Raman scattering (SERS), laser-induced breakdown spectroscopy (LIBS), and imaging techniques (visible light (VIS) imaging, NIRS imaging, HSI technology, terahertz imaging) with machine learning algorithms in the detection of pesticide residues in fruits and vegetables. It also explores the huge challenges faced by the application of spectroscopic and imaging technologies combined with machine learning algorithms in the intelligent perception of pesticide residues in fruits and vegetables: the performance of machine learning models requires further enhancement, the fusion of imaging and spectral data presents technical difficulties, and the commercialization of hardware devices remains underdeveloped. This review has proposed an innovative method that integrates spectral and image data, enhancing the accuracy of pesticide residue detection through the construction of interpretable machine learning algorithms, and providing support for the intelligent sensing and analysis of agricultural and food products.

## 1. Introduction

Pesticide residues in fruits and vegetables have always been one of the key challenges in food safety. While the widespread use of pesticides has effectively increased crop yields and quality, residual pesticide components can pose potential hazards to human health and the environment. Traditional detection methods, such as gas chromatography-mass spectrometry (GC-MS) and liquid chromatography-mass spectrometry (LC-MS), despite demonstrating high sensitivity and analytical accuracy, these methods are constrained by inherent limitations including operational complexity, time-intensive detection processes, substantial cost burdens, and reliance on specialized technical expertise [1,2,3]. Therefore, developing rapid, non-destructive, and efficient pesticide residue detection technologies holds significant scientific importance and practical value.

In practical pesticide detection applications, it is necessary to comprehensively consider performance metrics such as prediction correlation coefficient (R^2^_P_), accuracy, precision, sensitivity (or recall), root mean square error of prediction (RMSEP), ratio of performance to deviation (RPD), and F1-score [4]. R^2^_P_ is commonly used to determine the prediction accuracy of models for prediction datasets, which establish the relationship between instrument response and pesticide concentration. However, a high R^2^_P_ value does not necessarily guarantee that the model is suitable for all scenarios. Accuracy is the ratio of correctly classified samples (the number of true positives and true negatives) to the total number of samples in the evaluation dataset. It has limitations: if the dataset is imbalanced, accuracy may be misleading. Precision assesses the reliability of a model’s positive predictions for pesticide presence, i.e., the proportion of samples predicted to contain pesticides that are truly positive. Sensitivity measures how many truly pesticide-contaminated agricultural products the model can identify. RESEP is used to evaluate the predictive accuracy of a model on an independent test set, measuring the deviation between predicted values and actual values. RPD can reflect the strength of a model’s predictive capability relative to the inherent variability of the data, serving as a standardized performance metric. The F1-score is the harmonic mean of precision and recall [5]. However, not all studies use these indicators to describe the performance of the models, which to a certain extent, hinders the comparison of the advantages and disadvantages of the models.

Recently, machine vision technology has made significant progress in the detection of pesticide residues in fruits and vegetables. By integrating algorithms, this technology can quickly identify subtle changes caused by pesticide residues, enabling automated detection of fruits and vegetables, especially suitable for large-scale on-site testing [6,7]. Spectroscopy and imaging techniques complement each other in the detection of pesticide residues in fruits and vegetables. Spectroscopy can accurately capture chemical information from fruits and vegetables. Different pesticide residues cause specific changes in spectral characteristics. Imaging technology, on the other hand, can vividly display details such as the appearance, color, and texture of fruits and vegetables. The combination of both technologies achieves comprehensive detection from chemical information to physical appearance [8,9,10].

In addition to traditional machine learning, spectral techniques combined with deep learning algorithms, such as convolutional neural networks (CNNs, a neural network specifically designed for grid-structured data such as images and time-series signals) and generative adversarial networks (GANs, an unsupervised generative model that learns data distributions through an adversarial training framework, generating new samples with statistical properties similar to real data), can automatically extract complex features, optimize detection models, and significantly improve detection accuracy [11,12]. Tan et al. [13] used hyperspectral imaging (HSI) technology combined with GANs to predict pesticide residue levels in cantaloupe, with good detection results (R^2^_P_, RMSEP, and RPD were 0.8781, 0.6962, and 2.7882). Furthermore, it was also reported that terahertz spectroscopy combined Wasserstein GANs with residual networks (WGANs-ResNet) was used for pesticide detection, achieving an accuracy rate of 91.4% [14].

**Figure 1:**
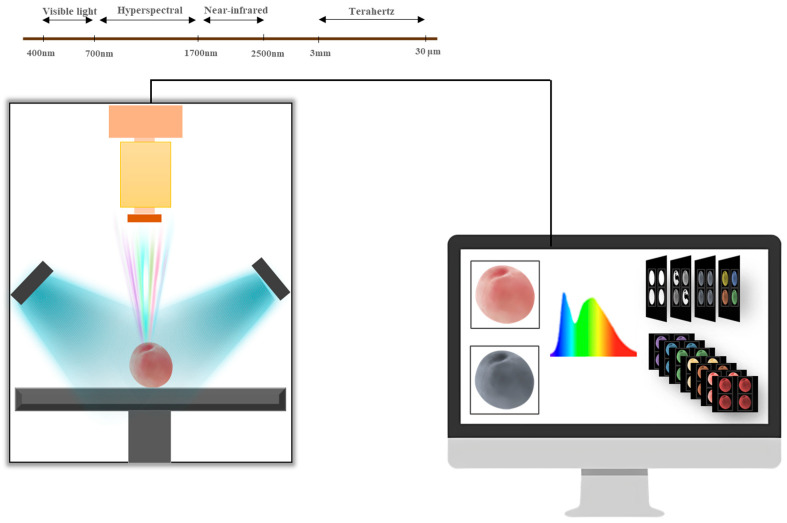
Scheme of the spectral analysis system (VIS imaging, NIRS imaging, HSI imaging, terahertz time-domain imaging) for the detection of agricultural and food products with spectral data and imaging data.

At present, multi-modal data fusion technology has further improved the accuracy and efficiency of detection by integrating different types of spectral and image information [15]. Moreover, with the continuous optimization of hardware equipment and improvement of algorithm models, these technologies could achieve broader applications. Herein, this review aims to summarize the advancements in the integration of spectroscopic techniques (near-infrared spectroscopy (NIRS), HSI, Raman spectroscopy) and imaging technologies (visible light (VIS) imaging, NIRS imaging, HSI, terahertz imaging) with machine learning for pesticide residue detection in fruits and vegetables (Figure 1). It focuses on analyzing the current challenges in research and looks forward to the future development trends of spectral/imaging technology combined with machine learning algorithms in the field of agricultural products. This review is expected to provide new insights into the quality, safety, and intelligent innovation of fruits and vegetables.

## 2. Spectral Techniques

**Figure 2:**
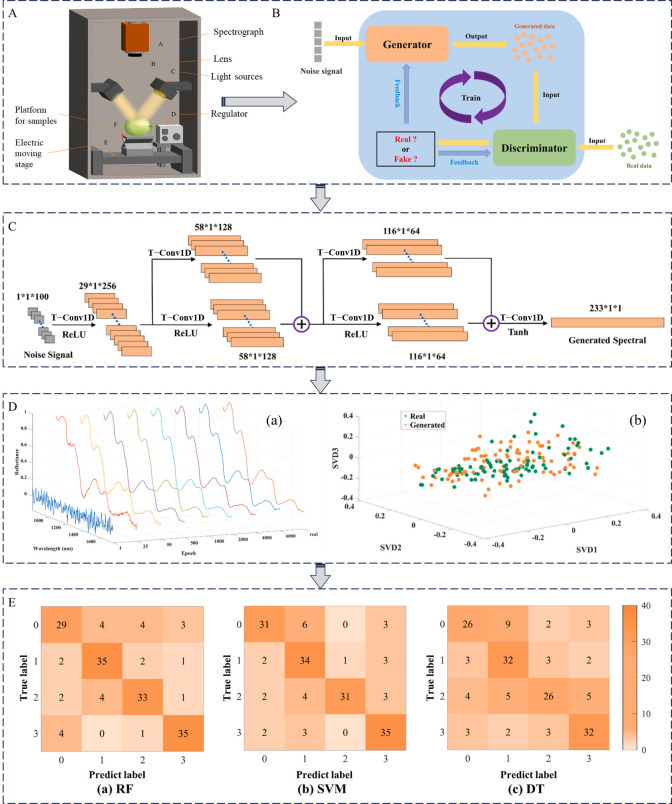
A typical flow chart of spectroscopy technology combined with machine learning for pesticide residue detection [16]. (**A**) Scheme of spectrometer; (**B**) The architecture of generative adversarial networks to obtain the spectral datasets; (**C**) The improved generator; (**D**) The spectrum generated by improved generative adversarial networks (a) and visualization of generated spectra (b); (**E**) The confusion matrix of classification results on spectral dataset.

Spectral technology (such as NIRS, Raman, etc.) captures the characteristic light absorption, scattering, or vibration information of pesticide and matrix molecules in fruits and vegetables, forming spectral signals that reflect the chemical composition of substances. Machine learning (such as preprocessing, feature extraction, pattern recognition, etc.) is used to denoise the original spectrum, eliminate matrix interference, and extract feature variables related to pesticide residues. Then, by establishing calibration models (such as partial least squares regression (PLSR), a commonly used multivariate statistical analysis method, especially when dealing with data with multicollinearity), support vector machine (SVM, a classic supervised learning algorithm primarily used for classification and regression tasks), etc.), the quantitative or qualitative correlation between spectral signals and pesticide residue levels is achieved, thereby enabling rapid and non-destructive detection of pesticide residues in fruits and vegetables. Spectral technology has demonstrated significant advantages such as high efficiency, rapidity, and non-destructiveness in the field of pesticide residue detection and has become one of the important detection methods in this field. Figure 2 shows a typical flow chart of spectral technology combined with machine learning for pesticide residue detection.

### 2.1. Near Infrared Spectroscopy

NIRS boasts advantages such as fast detection speed, no need for chemical reagents, non-destructive testing, and ease of operation. By integrating machine learning methods like PLS, a supervised multivariate statistical method used to model the linear relationship between independent variables and dependent variables, artificial neural networks (ANNs, a biologically inspired computational model composed of an input layer, hidden layer, and output layer), and CNNs, NIRS has been successfully applied to the detection of various pesticide residues, such as organophosphates and carbamate pesticides [12].

NIR diffuse reflectance spectroscopy (NIRDRS) (11,000–4000 cm^−1^) combined with PLSR was utilized to develop a non-destructive approach for detecting boscalid (0.03–26.94 mg/kg) and pyraclostrobin (0.22–6.89 mg/kg) residues in strawberries. Validated against QuEChERS-LC-MS/MS, the model demonstrated reliable quantitative performance (boscalid: RPD = 2.28, R^2^_P_ = 0.93, pyraclostrobin: RPD = 2.31, R^2^_P_ = 0.83). The association between spectral features and pesticide concentrations was established without dependence on specific absorption bands. Instead, latent information within the multi-component matrix was extracted through chemometrics. This technology can be employed as a rapid preliminary screening tool for conventional detection methods, significantly reducing associated costs and time [17]. Xue et al. [18] employed NIRDRS combined with particle swarm optimization (PSO) to select feature wavelengths within the 350–1800 nm range for PLS model development. Quantitative detection of dichlorvos residues on navel orange surfaces was achieved at concentrations (1:800 and 1:1000 *v*/*v*) below the recommended dosage (1:500 *v*/*v*). The optimized model (R^2^_P_ = 0.8735, corrected correlation coefficient (R^2^_C_ = 0.8641), RMSE of cross-validation (RMSECV = 0.9023), RMSEP = 0.8755) demonstrated significantly enhanced predictive accuracy compared to the full-spectrum PLS model (R^2^_P_ = 0.7853, R^2^_C_ = 0.8783, RMSECV = 0.8569, RMSEP = 1.1670). Furthermore, NIRDRS (348.45–1141.34 nm) coupled with a modified 1D-CNN algorithm was demonstrated to enable non-destructive detection of pesticide residues on Hami melon surfaces. A four-class classification was performed for water and three pesticides (chlorothalonil, imidacloprid, and pyraclostrobin at 1:1000 dilution), where the model incorporated multi-scale convolutional kernels (3/5/7) and feature fusion structures to achieve 95.83% test-set accuracy, outperforming conventional PLS discriminant analysis (PLS-DA) (88.33%) and SVM (85.83%). Particularly in binary classification (pesticide presence/absence), 99.17% accuracy with 100% true negative rate was attained, though spectral similarity between imidacloprid and pyraclostrobin led to misclassification [19]. Adding attention mechanism modules to 1D-CNNs could effectively enhance model efficiency and computational performance [19,20,21], further demonstrating the high efficiency and accuracy of NIRS combined with deep learning in fruit pesticide residue detection.

**Figure 3:**
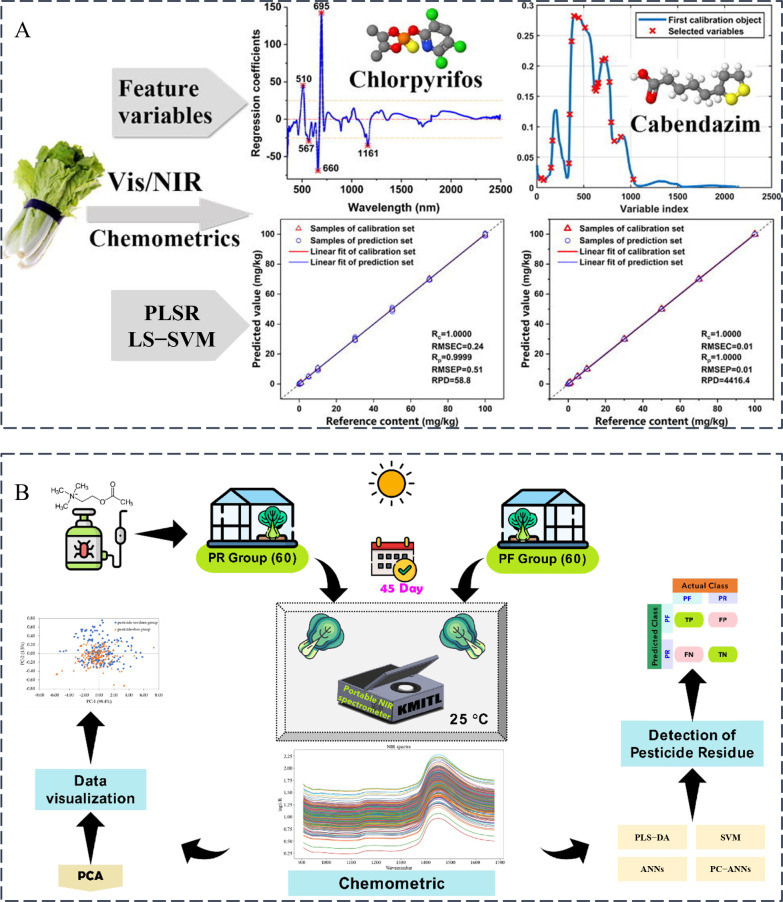
(**A**) Spectral analysis of pesticide residues in Chinese cabbage by VIS/NIR spectroscopy [22]; (**B**) Identification of chlorpyrifos residues on Chinese cabbage by combining NIRS with PLS-DA, SVM, ANNs, and principal component ANNs [24].

NIRS is also widely used in the detection of pesticide residues in vegetables. Rapid detection of chlorpyrifos (0.1–100 mg/kg) and carbendazim (0.1–100 mg/kg) residues in cabbage was achieved using NIRDRS (400–2500 nm) coupled with chemometric methods. Through analysis of spectral absorption characteristics and feature wavelength selection, combined with LS-SVM modeling, 100% classification accuracy was attained for identifying exceedance thresholds (chlorpyrifos > 0.1 mg/kg, carbendazim > 5 mg/kg). For quantitative analysis, carbendazim demonstrated exceptional performance (full-spectrum LS-SVM: R^2^_P_ = 1.000, RMSEP = 0.01 mg/kg, 99.94% recovery at 0.1 mg/kg with relative standard deviation (RSD = 6.99%)), whereas chlorpyrifos showed higher variability (RSD = 17.34% at 0.1 mg/kg). This approach proved faster and non-destructive compared to conventional chromatography, though optimization for low-concentration chlorpyrifos detection remained necessary (Figure 3A) [22]. In subsequent research, NIRDRS (500–2400 nm) integrated with chemometrics enabled non-destructive monitoring of three pesticides (abamectin, dichlorvos, chlorothalonil, abamectin, and dichlorvos were prepared into solutions with five concentration gradients: 0.25, 0.5, 0.75, 1, and 2 mg/kg, while chlorothalonil was formulated into solutions with concentrations of 2, 5, 10, 15, and 20 mg/kg) on cauliflower surfaces. Feature wavelengths selected by regression coefficient (RC), successive projections algorithm (SPA), and competitive adaptive reweighted sampling (CARS) were employed to develop PLS-DA and LS-SVM classification models. RC-LS-SVM achieved a detection accuracy of 98.33% and an F1-score of 98.40% for abamectin, SPA-LS-SVM yielded a detection accuracy of 95.00% and an F1-score of 95.00% for dichlorvos, and CARS-PLS-DA showed a detection accuracy of 93.33% and an F1-score of 93.45% for chlorothalonil. Models effectively identified exceedance cases (e.g., abamectin > 0.5 mg/kg), though spectral overlap or degradation in high-concentration samples caused misclassification [23]. Furthermore, NIRDRS (908–1676 nm) combined with PLS-DA, SVM, and principal component ANN (PC-ANN) algorithms was implemented for qualitative classification of chlorpyrifos (2 mL/L) residues in pak choi. All evaluated models demonstrated 100% performance metrics (accuracy, precision, recall, F1-score) on unknown datasets (Figure 3B) [24]. For tomato pesticide analysis, NIRDRS with PLS-DA successfully detected chlorpyrifos at maximum residue levels (MRL = 10 mg·kg^−1^). The results revealed that the calibration and validation sets achieved accuracies of 90% and 91.66%, respectively, accompanied by high true positive and true negative rates. Specifically, within the calibration set, the correct classification rate exceeded 90% for the 40 healthy samples and 97% for the 72 unhealthy samples. In the prediction set, the correct classification rate was over 80% for the 18 healthy samples and 75% for the 30 unhealthy samples. Compared to GC methods, NIRDRS provided comparable accuracy while offering non-destructive, rapid analysis without extensive sample preparation [25].

### 2.2. Hyperspectral Data Analysis 

The combination of HSI and deep learning can automatically extract complex spectral features, optimize model performance, improve detection accuracy, and generalize the model. The study has demonstrated the feasibility of non-destructive, rapid detection of three pesticide residues (acetamiprid, malathion, and lambda-cyhalothrin at a 1:1000 concentration) on Hami melon surfaces using short-wave HSI (SWIR-HSI) combined with an enhanced deep convolutional GANs data augmentation technology. The proposed technology effectively addressed spectral oscillation issues through the introduction of a dual-branch architecture within the generator. The quality of the generated high-quality SWIR-HSI data was confirmed by 1-nearest neighbor assessment (achieving 64.51% accuracy) and singular value decomposition validation. Results showed that this approach significantly enhanced detection performance under small-sample conditions (originally only 480 data points). The classification accuracy of SVM was increased from 81.88% on the original data to 93.13% (an 11.25% improvement) after adding 500 generated samples per class. Decision tree and random forest (RF) models also showed improvements of 13.13% and 7.50%, respectively. Compared to traditional destructive and high-cost chromatographic detection methods, this research provides an efficient and non-destructive alternative approach [16].

Another study utilized reflectance spectroscopy-based HSI to reduce matrix interferences in red dates, employing preprocessing methods such as Savitzky–Golay (SG) smoothing and Kubelka–Munk (K-M) transformation. Combined with chemometric methods including PLSR, linear discriminant analysis (LDA), and support vector regression (SVR), the detection of three pesticide residues (chlorpyrifos, imidacloprid, and pyridaben, all are configured at a ratio of 1:500) on the surface of red dates was conducted. For the discriminant calibration model of pesticide types, the overall recognition rate was 96.43% in the calibration set and 91.7% in the prediction set. For the qualitative discrimination of chlorpyrifos concentration, the accuracy of the calibration set was 72.68% and that of the prediction set was 50%. In the quantitative models, at the characteristic wavelengths, RMSE of calibration (RMSEC) and RMSEP for chlorpyrifos were 0.0025 and 0.0031, respectively, and for imidacloprid, the RMSEC and RMSEP were 0.00032 and 0.00033, respectively. Moreover, the stability of the model based on characteristic wavelengths was superior [26].

HSI technology is also widely used in the detection of pesticide residues on vegetables. HSI technology (950–1666 nm) combined with the standard normal variate PLS (SNV-PLS) model enables accurate and non-destructive detection of emamectin benzoate and indoxacarb residues in cauliflower. The residual concentrations of the two pesticides in cauliflower at different time points are as follows: 2.70 mg/kg and 14.79 mg/kg on day 0, 1.24 mg/kg and 9.00 mg/kg on day 1, 1.36 mg/kg and 9.00 mg/kg on day 3, 0.74 mg/kg and 7.66 mg/kg on day 5, 0.38 mg/kg and 6.95 mg/kg on day 7, 0.30 mg/kg and 6.24 mg/kg on day 9. The results showed that the optimal SNV-CARS-PLS model achieved a discrimination accuracy of 96.88%, an RMSE of 0.2648, and a recognition rate of 100%, indicating its great potential in pesticide residue detection [27]. In another investigation focusing on the detection of glyphosate and bifenthrin pesticides, HSI (900–1700 nm) was employed to collect data from 5 fresh white tea leaf samples with pesticide residues, and a one-dimensional CNN model was utilized for feature extraction. Results indicated that the SG-CARS-1D-CNN model achieved the highest accuracy [28]. Additionally, HSI (400–1000 nm) integrated with 1D-CNNs and channel attention mechanism (CAM) was developed for the non-destructive detection of four pharmaceutical residues (gibberellin, indoleacetic acid, oxytetracycline, and procymidone, prepared at commercially recommended concentrations) in mung bean sprout cotyledons. Following region-of-interest extraction via Otsu threshold segmentation and preprocessing with SG smoothing and SNV, three feature extraction methods (principal component analysis (PCA, an unsupervised linear dimensionality reduction technology that transforms original correlated variables into principal components ordered by decreasing variance), iteratively retained informative (IRF), SPA) were compared. The IRF method was found to effectively screen characteristic wavebands concentrated within the spectral differentiation region of 520–700 nm, enhancing the performance of traditional models. Results demonstrated that traditional machine learning models (PSO-SVM and extreme learning machine) attained a maximum accuracy of only 92.6%, with a notable misclassification rate exceeding 10% observed between oxytetracycline and indoleacetic acid groups. In contrast, the 1D-CNNs-CAM model employed channel attention to adaptively weight critical spectral features, achieving an overall accuracy of 96.3% with full-spectrum input while significantly reducing contiguous misclassified regions. Its performance was comparable to the state-of-the-art cardiovascular magnetic resonance CNN model (96.5%), while demonstrating superior capability in gibberellin group identification (93% accuracy) and achieving 100% accuracy in sample-level voting validation. This approach provides an efficient solution for rapid pharmaceutical residue detection in bean sprouts [29].

In the detection of pesticide residues in vegetables, the application of HSI technology combined with deep learning methods continues to expand. Rapid and non-destructive quantitative detection of mixed pesticide residues, specifically beta-cypermethrin (8.06–18.96 mg/kg) and dimethoate (17.25–60.91 mg/kg), in lettuce leaves was achieved using HSI technology within the 431–962 nm range. After preprocessing spectral data via SNV, 44 wavelengths for beta-cypermethrin were selected using CARS, while 124 wavelengths for dimethoate were identified through RF recursive feature elimination (RF-RFE). These selected wavelengths were then further optimized to 14–16 key wavelengths using the SPA for secondary refinement. By integrating the LS-SVR for modeling, the CARS-SPA-LS-SVR model ultimately yielded R^2^_P_ of 0.8890 and RMSEP of 0.0182 mg/kg for beta-cypermethrin, whereas the RF-RFE-SPA-LS-SVR model achieved R^2^_P_ of 0.9386 and extremely low RMSEP of 0.0077 mg/kg for dimethoate. Compared with traditional chromatographic methods (GC-MS/LC-MS), high precision was maintained (with RMSEP reduced by over 58%) while the model was significantly simplified (wavelengths reduced from 450 to 14–16) [30]. This study further confirmed the high efficiency and practicality of integrating HSI technology with deep learning in pesticide residue detection in vegetables.

### 2.3. Raman Spectroscopy

**Figure 4:**
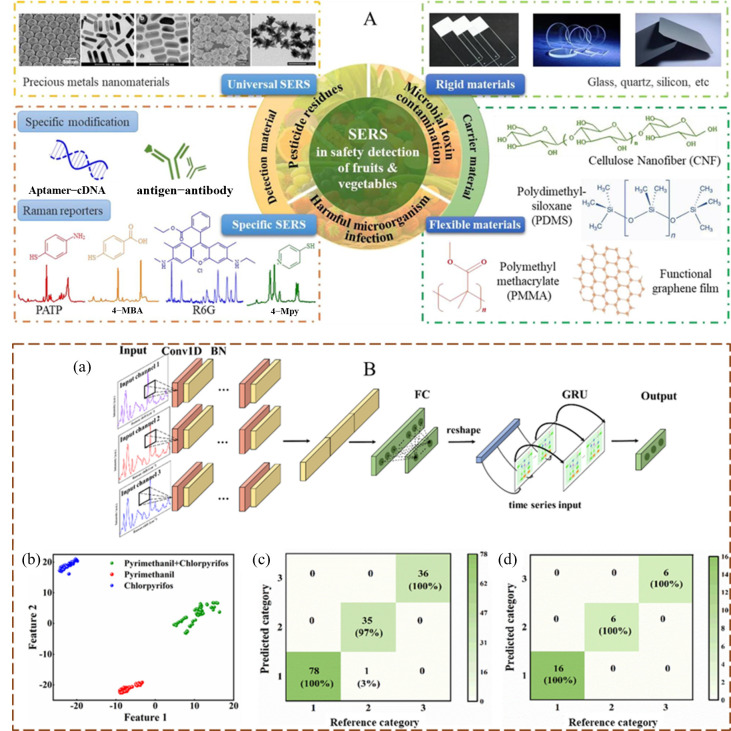
(**A**) The binding mechanism of detection materials and carrier materials for the identification of pesticide residues in fruits and vegetables using SERS [31]; (**B**) (a) The MC-CNNs-GRU discrimination model for pesticide/fungicide residues; (b) The t-SNE analysis results of the MC-CNNs-GRU model; (c) The confusion matrix of the training set and (d) the test set [32].

Raman spectroscopy combined with algorithms can automatically identify complex spectral features, enhancing the accuracy and efficiency of detection. The integration mechanism between the detection materials and carrier materials utilized in this technology for pesticide residue analysis in fruits and vegetables is schematically illustrated in Figure 4A [31]. Studies have integrated surface-enhanced Raman scattering (SERS) technology (within the range of 200–1700 cm^−1^) with deep learning-based spectral analysis, aiming to detect mixed residues of chlorpyrifos (10^−3^–10^−7^ mol/L) and pyrimethanil (10^−3^–10^−7^ mol/L) on the surfaces of fruits such as apples and strawberries. A multi-channel CNN network-gated recurrent unit (MC-CNNs-GRU, GRU is a variant of recurrent neural networks specifically designed for processing sequential data) classification model (Figure 4B) was employed to effectively extract sequential and spatial features from the spectra. The results indicated that the optimized classification model achieved an accuracy of 99% even when there are significant variations in the mixing ratios of the pesticide and fungicide. In individual detection, the limits of detection (LODs) of chlorpyrifos and pyrimethanil on fruit surfaces are 2 × 10^−4^ mg/cm^2^ and 1.2 × 10^−4^ mg/cm^2^, respectively. In mixed detection, the LODs of chlorpyrifos and pyrimethanil are 4.2 × 10^−4^ mg/cm^2^ and 2.4 × 10^−4^ mg/cm^2^, respectively [32]. A separate investigation utilized SERS (400–2500 cm^−1^) based on silver nanoparticle colloids coupled with chemometric modeling to quantify carbendazim residues in apple juice. The analytical approach exhibited a linear response across the concentration range of 0.1–10 mg/L, with the LOD of 0.01 mg/L. The findings revealed that the bootstrapping soft shrinkage PLS method, following variable selection, delivered superior prediction accuracy; the final model achieved a high prediction accuracy (R^2^_P_ = 0.992) [33]. This indicates that the combination of SERS technology with deep learning algorithms can further improve the sensitivity and specificity of detection, providing an alternative method for rapid pesticide residue testing in fruits.

By integrating Raman spectroscopy with deep learning algorithms, researchers have achieved highly sensitive detection of pesticide residues in various vegetables. SERS technology (400–2400 cm^−1^) combined with chemometric methods was developed for the qualitative identification and quantitative determination of four benzimidazole (BMZ) residues in maize. For qualitative analysis, the PLS-DA model demonstrated optimal performance, achieving a recall rate of 99.17% and successfully differentiating the four BMZs. In quantitative analysis, the LODs for each BMZ were determined using an SVR model as follows: 0.055 mg/L for carbendazim, 0.056 mg/L for benomyl, 0.067 mg/L for thiophanate-methyl, and 0.093 mg/L for thiabendazole. The average recoveries for the four BMZs ranged between 85.6% and 107.5%. Compared with HPLC, the developed method showed no significant differences in results (*p* > 0.05) [34]. In separate research, SERS (200–3300 cm^−1^) coupled with machine learning was employed for the detection of thiabendazole, carbendazim, and chlorpyrifos on tomato surfaces, achieving LODs of 20 ng/cm^2^, 36 ng/cm^2^, and 80 ng/cm^2^, respectively. Significant enhancement in the uniformity and sensitivity of the SERS signals was achieved through parameter optimization. This approach offered significantly simplified pretreatment procedures and high sampling efficiency (RSD < 9%) compared to traditional HPLC methodology [35]. In the future, with the development of portable Raman spectrometers and the optimization of deep learning models, this technology is expected to enable rapid and real-time pesticide residue detection in field and market environments.

### 2.4. Laser-Induced Breakdown Spectroscopy

Laser-induced breakdown spectroscopy (LIBS) technology has demonstrated significant potential in pesticide residue detection due to its advantages of being fast, non-destructive, and requiring no complex sample pretreatment. LIBS offers advantages in rapid on-site testing, enabling not only the identification of toxic substances on fruit surfaces but also semi-quantitative detection capabilities [36]. The rapid measurement of pesticide residues in beet leaves by LIBS was carried out, and the feasibility of grouping samples according to pesticides was verified by LDA, with an error rate of less than 9.5% [37].

LIBS-HSI integrated with machine learning methods was successfully applied for the rapid detection of thiophanate-methyl residues in mulberries across a concentration range of 0–5.0 × 10^−3^ g·mL^−1^. Elemental spectral information within the 270–850 nm range was acquired by LIBS, while molecular bond vibration data were collected across the 400–1000 nm and 900–1700 nm spectral bands using dual HSI systems. The fused spectral data were subjected to dimensionality reduction via PCA, and a quantitative model was established based on PLSR. Following variable optimization by CARS, this model demonstrated excellent predictive performance on the independent validation set (R^2^_P_ = 0.921, RPD = 2.585, RMSEP = 7.09 × 10^−4^), effectively confirming the feasibility of the LIBS-HSI fusion approach for detecting this residue [38]. Furthermore, silver nanoparticle-enhanced LIBS (NELIBS) was employed, which significantly improved the detection sensitivity for chlorpyrifos pesticide (48–240 mg/L) and cadmium (3–60 ng/g) in fruits and vegetables. The LOD for chlorpyrifos was reduced to 0.009–0.029 mg/kg (two orders of magnitude lower than that of traditional LIBS), and the LOD for cadmium reached 1.6 ng/g (outperforming by three orders of magnitude). Specificity was confirmed through characteristic peak analysis and blank control [39].

## 3. Spectral Imaging Technology

**Figure 5:**
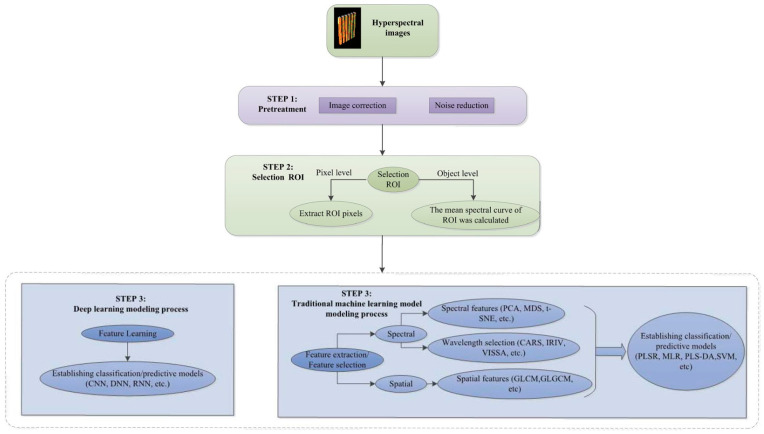
Scheme of processing for food hyperspectral image datasets [40].

Spectral imaging systems, including VIS imaging, NIRS imaging, HSI, and terahertz time-domain imaging, acquire spectral images of the surface of fruits and vegetables. By processing and analyzing high-dimensional spectral data using machine learning algorithms, these systems can effectively identify and quantify pesticide residues (Figure 5). The integration of spectral imaging systems with machine learning algorithms enables rapid, non-destructive, and low-cost pesticide residue analysis, providing efficient approaches for the safety testing of agricultural products.

### 3.1. Visible Light Imaging Technology

VIS combined with HSI technology can identify pesticide residues of Jiatu, Huiying, and Xisongke (prepared at ratios of 1:4000, 1:6000, and 1:24,000, respectively) on grape surfaces. This technology works by collecting images of grape surfaces and integrating them with image processing and analysis algorithms. Through the establishment of a correlation model between spectral characteristics and concentrations, combined with preprocessing, feature selection, and regression algorithms, accurate mapping from images to concentrations was achieved [12,41]. The combination of VIS imaging technology and the improved YOLO v3 algorithm has been applied to detect pesticide residues on apple surfaces. The results showed that this method can achieve high-precision identification in complex orchard environments, with an accuracy rate of 95% [42].

Additionally, another study employed VIS-HSI technology to detect defects in Prunus tomentosa fruits. Based on the full spectrum, the PLS-DA and back propagation neural network (BPNN) models achieved detection accuracies of 83.81% and 85.71%, respectively. After screening characteristic wavelengths using CARS, the accuracy of the BPNN model was improved to 90.47%. Finally, based on the principal component images after PCA dimensionality reduction, the CNN model achieved a classification accuracy of 93.33%, which was significantly superior to the 83.81% of the BPNN model. This demonstrates the superiority of CNN models in defect detection using hyperspectral images [43]. Moreover, VIS imaging technology utilized distinctive features from multi-sensor acquisitions, preserving complementary information from source images during fusion while leveraging redundant data to enhance the robustness of fused images [44]. By combining VIS imaging technology with deep learning algorithms to detect pesticide residues in Chinese cabbage, the results showed that this method can effectively distinguish between mild and severe pesticide residues, laying the foundation for the development of portable pesticide residue detection devices [45].

### 3.2. Near Infrared Spectroscopic Imaging Technology

NIRS imaging technology combined with algorithms has made significant progress in the detection of pesticide residues in fruits. Li et al. [46] employed an NIRS imaging system to detect dichlorvos residues on orange surfaces and established a predictive model for dichlorvos residue levels, achieving R^2^_P_ of 0.832 in PLS analysis with a mean standard error of 1.3416. Some studies have also employed NIRS imaging to detect pesticide residues on navel oranges, revealing how different concentrations of pesticides change over time on the surface of navel oranges [47]. By integrating NIRS with image-based neural network features, the accuracy of “Dang shan” pear root rot can be greatly enhanced [48]. In the detection of pesticide residues in vegetables, relevant studies on NIRS imaging technology combined with machine learning methods are relatively scarce and need to be continuously expanded.

### 3.3. Hyperspectral Imaging Technology

When combined with deep learning, HSI technology can automatically extract complex spectral features, optimize model performance, and improve detection accuracy and generalization ability. However, the spectral cube data collected by HSI systems is relatively complex. Therefore, to accurately extract spectral data, relevant processing of the original image is required [49].

HSI technology combined with machine learning algorithms has made significant progress in pesticide residue detection on fruits. Residues of chlorpyrifos (100 ppm), carbendazim (100 ppm), and two mixed pesticides (100 ppm) in apples were effectively detected in a study by utilizing a CNN-based method combining machine vision and HSI technology. The results showed that when the training epoch was set to 10, the detection accuracy on the test set reached 90.09%, with an average image bandwidth detection accuracy of 95.35%. Compared to traditional algorithms, this method is faster, more cost-effective, and suitable for small-sample detection [50]. A non-destructive approach based on SWIR-HSI and 1D-CNNs enables rapid and accurate detection and identification of pesticide residues on garlic chives leaves. HSI were collected from chive leaves sprayed with pure water, single pesticides (λ-cyhalothrin, trichlorfon, phoxim, diluted with pure water at the recommended agricultural concentration of 1:500), and mixed pesticides, followed by preprocessing that incorporates improved mean filtering and the isolation forest algorithm for outlier detection and removal. The 1D-CNN model was constructed, achieving accuracies of 98.5%, 98.0%, and 97.9% on the training, development, and test sets, respectively. Additionally, the area under the receiver operating characteristic curve (AUC) for all categories exceeded 0.99, significantly outperforming traditional classification models such as k-nearest neighbors, LDA, naive Bayes, RF, and SVM [51]. In addition to pears and Hami melons, HSI technology has demonstrated strong application potential in detecting pesticide residues on other fruits such as navel oranges, peaches, and apples [10].

**Figure 6:**
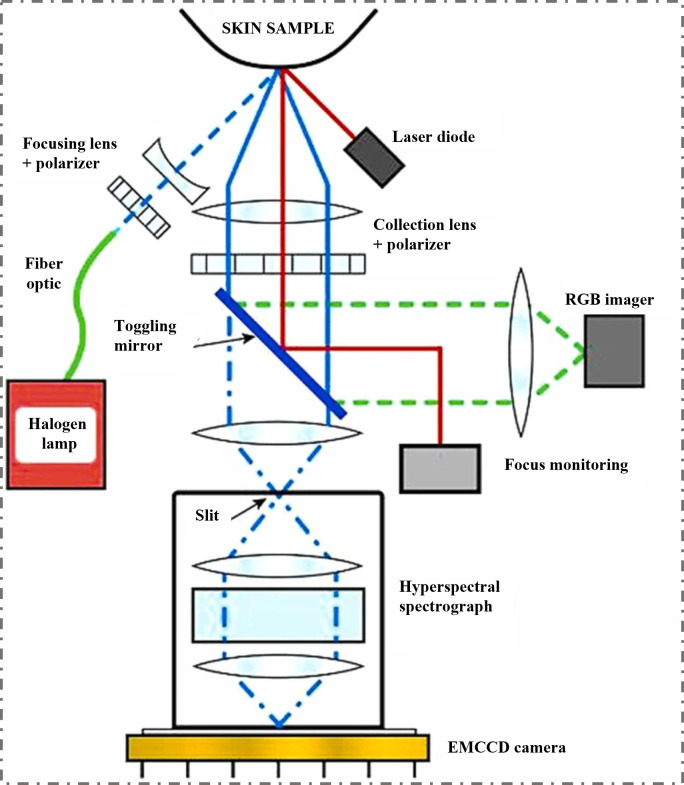
The operation of the HSI imaging sensor [56].

HSI technology is also widely used in the detection of pesticide residues in vegetables. Studies integrating hyperspectral images with algorithms have achieved rapid, non-destructive, in-situ quantification of foliar nitrogen content in soybeans. Findings confirm HSI’s efficacy for spatial prediction of nitrogen levels in soybean leaves [52]. Using HSI technology and predictive classification models, the feasibility of non-destructive pesticide residue detection methods for tomatoes was studied and compared with models based on images acquired from commercial cameras. The preliminary results confirmed the feasibility and effectiveness of HSI for detecting pesticide residues on tomatoes [53]. Whether olives have been sprayed with pesticide products was detected by an HSI-based method proposed in a study. Under controlled laboratory conditions, the method achieved an accuracy rate of over 80% for most pesticides [54]. It has been demonstrated that combining HSI with spectral preprocessing and feature extraction algorithms, and optimizing SVM models using grid search, significantly enhanced the performance of pesticide residue detection in tobacco, achieving model accuracy rates exceeding 84% [55]. Furthermore, by integrating HSI and RGB images, the location of pesticide application and its concentration in specific areas can be determined (Figure 6), while further improving detection accuracy using spectral and image information [56].

### 3.4. Terahertz Imaging Technology

Most pesticide molecules have specific absorption frequencies in the terahertz band. By comparing the vibration frequencies and characteristic absorption peaks of different pesticides, new ideas for qualitative identification of pesticide residues have been provided [57]. Terahertz imaging technology combined with machine learning has made significant progress in detecting pesticide residues on fruits. Terahertz imaging technology combined with machine learning was employed to detect carbendazim (1–6 ppm trace concentration) pesticide residues on apple peels, integrating theoretical simulations with experimental measurements in some studies. The results demonstrated that terahertz imaging technology combined with deep learning could rapidly and accurately detect pesticide residues in fruits. The constructed regression model exhibits high prediction accuracy (R^2^_P_ = 0.9750) and a low LOD (1.12 ppm) [58].

**Figure 7:**
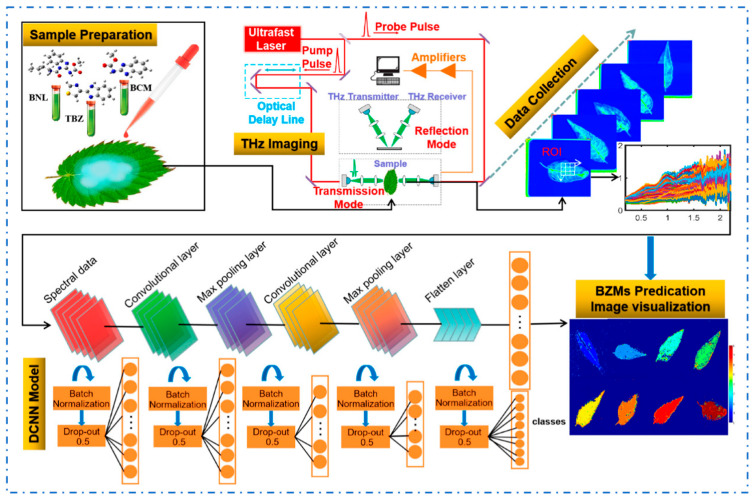
The flowchart of detecting multiple benzimidazole pesticide residues in Toona sinensis leaves using terahertz imaging and deep learning [59].

Terahertz imaging technology was also widely used in the detection of pesticide residues on vegetables. By combining high-throughput terahertz imaging with deep learning algorithms, it can rapidly identify trace residues of benzimidazole compounds (benzoyl, carbendazim, and thiram, unified trace concentration: 10 mg·L^−1^) on Chinese toon leaves, with prediction accuracies of 100%, 94.51% and 96.26% [59] (Figure 7). Utilizing terahertz imaging for spatial localization and quantitative analysis of carbendazim residues in tea leaf matrices, this research validated terahertz spectroscopy as an effective detection methodology for carbendazim contaminants [60].

## 4. Challenges and Prospects

**Figure 8:**
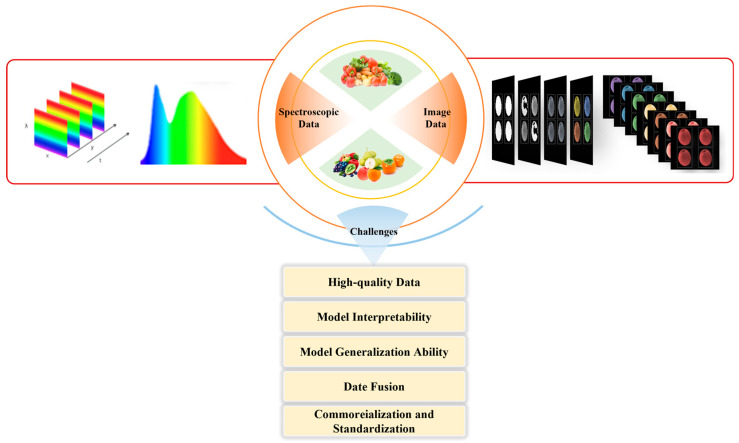
The integration of spectral and image data with machine learning algorithms for pesticide residue identification in fruits and vegetables: challenges and future prospects.

One-dimensional spectral data provides characteristic information pertaining to food components and molecular vibrations. The processing of spectral data facilitates the rapid detection of compositional changes in food, thereby enabling the identification of pesticide residues. This method circumvents the complexities associated with traditional chemical analyses. In contrast, two-dimensional spectral images, such as Raman spectroscopy, fluorescence spectral images, and HSI, convey more intricate multi-level characteristic information about food samples. Accurately identifying subtle differences in spectral images can effectively differentiate between intrinsic and extrinsic components of food. The integration of spectral and image data with machine learning algorithms yields exceptionally high detection efficiency and accuracy for pesticide residue identification in fruit and vegetable products. There are difficulties in data annotation for spectral data and image data. Image data contains more redundant components, such as noise, which will affect the accuracy of the model. In addition, the fusion modeling of spectral data and image data increases the complexity of the model [61]. The interpretability and generalization ability of the model are also challenges faced in the detection of pesticide residues in fruits and vegetables using machine vision (Figure 8).

### 4.1. Challenges in Detecting Low-Concentration Pesticide Residues

The detection of low-concentration pesticide residues in fruits and vegetables based on spectroscopy or imaging faces significant challenges due to interference from high-abundance matrices. The matrix components, present at concentrations far exceeding those of the pesticides, generate intense background signals that overlap with the characteristic pesticide signatures. This overlap, compounded by spectral drift induced by intermolecular interactions, obscures the distinctive fingerprint features of the pesticides. The inherently low concentration of pesticides results in signal intensities that approach or fall below the level of interference, leading to an extremely low signal-to-noise ratio. Furthermore, nonlinear signal attenuation amplifies quantification errors. Dynamic interactions between the matrix and pesticides can also alter the pesticides’ optical properties, and this variability is matrix-specific, further complicating the interpretation [62,63,64].

Simultaneously, the high-dimensional nature of spectral/imaging data contains substantial redundant information. Low-concentration pesticide signals are easily misidentified as noise or artifacts, causing traditional feature selection methods to fail. Models require high sensitivity yet struggle to cope with matrix fluctuations, creating a conflict between robustness and achievable LOD. Spatial heterogeneity in imaging data further exacerbates generalization difficulties. The limited dynamic range of instruments makes it difficult to capture both strong (matrix) and weak (pesticide) signals simultaneously, often leading to truncation or loss of the weak target signals. These issues collectively constitute major obstacles to effective detection [65,66,67].

Addressing these problems requires a multi-dimensional approach encompassing signal enhancement, interference separation, model optimization, and instrument improvement. Advanced preprocessing algorithms (e.g., adaptive iteratively reweighted PLS) can be leveraged to suppress the matrix background [68,69]. Deep learning techniques, particularly those incorporating attention mechanisms, can be employed to extract subtle low-concentration features. Transfer learning can enhance model adaptability to variations in matrix composition. Concurrently, developing detectors with high dynamic range is crucial for the simultaneous capture of signals spanning a wide intensity range [70,71].

Additionally, multi-modal data fusion (e.g., combining spectroscopic and imaging information) can provide complementary information. Molecular modification techniques, such as utilizing nanomaterials for SERS, offer pathways to boost the intensity of characteristic pesticide peaks [72,73,74]. Integrating these strategies aims to break through the bottleneck of detecting low-concentration pesticide signals, enabling precise and reliable residue detection.

### 4.2. High-Quality Data and Annotation

The variety of fruits and vegetables, their growing environments, and pesticide usage habits differ significantly across regions, adding complexity to the data. Existing machine learning models, after being trained in one region, often see a significant drop in detection accuracy when applied to other regions. Machine learning models, especially deep learning models, typically rely on features learned from training data for prediction. For example, if the training dataset only includes apples, the model may overfit the characteristics of apples while neglecting those of oranges. When the model is applied to orange detection, its generalization ability will be greatly reduced due to the inconsistency between the input data features and the training data [42].

In addition, the data annotation required for model training demands substantial experimental and computational resources, making it a labor-intensive and costly process [75]. Moreover, the annotation process is susceptible to subjective factors, leading to variations in results from different annotators, which can affect the accuracy of model training. Currently, the scarcity of accurate annotated data has become a bottleneck constraining the improvement of robustness in machine learning models [76].

### 4.3. Fusion Modeling of Spectral Data and Spectral Images

The integration of spectroscopy and imaging technologies in the detection of pesticide residues in fruits and vegetables faces numerous challenges. There are significant differences between spectroscopy and imaging techniques in terms of data acquisition frequency and resolution, which makes it difficult to match information during data fusion, leading to the loss of critical information. Image data and spectral data have different characteristics and dimensions [77,78]. Effectively fusing these with distinct features and dimensions, and extracting valuable information for pesticide residue detection, is one of the research difficulties. Existing data fusion algorithms are mostly feature-based, but they struggle to fully exploit the intrinsic connections between them in practical applications, resulting in suboptimal detection accuracy after fusion. Moreover, the quality of data obtained from different spectroscopic and imaging techniques varies, making it challenging to effectively integrate these disparate datasets. Complex real-world application scenarios further exacerbate this challenge [79]. Additionally, there are significant differences in data acquisition methods between imaging and spectroscopic instruments, which also pose numerous difficulties in integrating data into a single detection system.

### 4.4. Generalization Ability

Machine learning models have shown significant results in detecting pesticide residues in fruits and vegetables, but there are still many issues with model construction. During training, the model learns too much from the training data, leading to poor performance on new test data. This may be because the model has learned noise and specific details from the training data that are not common characteristics of pesticide residues [80]. To address overfitting, techniques such as data augmentation and regularization are often used [81], but their effectiveness varies depending on data characteristics and model structure, requiring continuous optimization.

In the construction of machine learning or deep learning models, future research should focus on innovative studies of model architecture to develop more efficient and generalizable models. For example, exploring the application of deep learning models based on Transformer architecture in pesticide residue detection for fruits and vegetables, leveraging its self-attention mechanism to better capture long sequence dependencies in data, thereby enhancing the model’s ability to identify pesticide residues in different scenarios. A high-resolution remote sensing (HRRS) image scene classification framework has been effectively developed by researchers through the integration of channel-spatial attention (CSA) mechanisms with vision transformer architectures, referred to as the CSA Transformer. The framework synergistically extracts channel-specific and spatial characteristics of HRRS imagery through coordinated feature learning from both CSA mechanisms and multi-head self-attention operations within transformer modules [82,83]. At the same time, by combining transfer learning and federated learning techniques, fine-tuning models trained in source domains with limited target domain data can reduce reliance on large-scale labeled datasets, which helps improve the model’s generalization performance.

### 4.5. Interpretability of Deep Learning Models

Deep learning has revolutionized the field of artificial intelligence by providing sophisticated models applicable to diverse domains ranging from image and speech recognition to natural language processing and autonomous driving [84]. However, deep learning models are often referred to as black boxes due to the lack of transparency and interpretability in their decision-making processes. This opacity hinders the ability to decipher the underlying logic of their decisions, thereby compromising model interpretability, accountability, and reliability [85,86].

The interpretability of deep learning models constitutes a vital research direction in the domain of agricultural product safety, specifically for pesticide residue analysis. By implementing appropriate explainable artificial intelligence (XAI) methodologies, these complex models can be rendered more transparent [87]. Enhanced interpretability not only improves trustworthiness but also facilitates broader adoption in food safety monitoring systems, ultimately contributing to the protection of public health.

### 4.6. Insufficient Commercialization

The development of hardware and the commercial deployment of machine learning models have rapidly advanced in the field of pesticide residue detection in fruits and vegetables. In recent years, the accuracy and stability of various portable devices have significantly improved, and detection models suitable for different scenarios have also been developed, capable of quickly processing large amounts of image and spectral data, performing well in identifying pesticide residues in fruits and vegetables. However, the reliability of commercial equipment in complex agricultural environments, such as high temperature and humidity, still needs optimization [88], while the high cost has become a core obstacle for commercialization, profoundly affecting market acceptance, profit models, and competitive dynamics. Additionally, the lack of standardization leads to training instability, performance fluctuations, and deployment failures, urgently necessitating end-to-end consistency through fixed preprocessing parameters, model design, and deployment pipeline integration [89,90].

In practical applications, spectroscopy-based detection technologies face multiple challenges, including drastic variations in ambient light, individual sample differences, unstable operating conditions, and background interference. Meanwhile, although deep learning models perform excellently under ideal laboratory conditions, their robustness is insufficient, making it difficult to adapt to extreme variations in real-world scenarios. The cost of acquiring the vast amounts of accurately labeled data needed to cover these variations is extremely high. Furthermore, the wide variety of pesticides, diverse residue forms, extremely low concentrations, and frequent presence as mixtures make the reliable identification and quantification of trace pesticide residue signals from complex matrices and noise a critical bottleneck, further raising the threshold for technology implementation [91,92].

In the future, integrating machine learning models with IoT systems to achieve comprehensive safety monitoring of fruits and vegetables from planting, harvesting, and transportation to sales will be a key focus. This will involve establishing more robust testing standards and norms. By leveraging interdisciplinary integration and in-depth exploration of practical application scenarios, we can promote the deployment of machine vision technology combined with algorithmic models, facilitating the development of portable equipment for real-world applications. This will help enhance the level of quality and safety assurance for agricultural products.

## Data Availability

No new data were created or analyzed in this study. Data sharing is not applicable to this article.
